# Fiber-Optic Magnetic Field Sensing Based on Microfiber Knot Resonator with Magnetic Fluid Cladding

**DOI:** 10.3390/s18124358

**Published:** 2018-12-10

**Authors:** Yuqi Li, Shengli Pu, Yongliang Zhao, Tianjun Yao

**Affiliations:** 1 College of Science, University of Shanghai for Science and Technology, Shanghai 200093, China; 162281923@st.usst.edu.cn (Y.L.); 162281927@st.usst.edu.cn (Y.Z.); 162281924@st.usst.edu.cn (T.Y.); 2 Shanghai Key Laboratory of Modern Optical System, University of Shanghai for Science and Technology, Shanghai 200093, China

**Keywords:** microfiber knot resonator, magnetic fluid, quality factor, finesse

## Abstract

A kind of all-fiber magnetic field sensing structure is proposed and demonstrated here. The sensing element includes a microfiber knot resonator (MKR) cladded with magnetic fluid (MF). The low-index MgF_2_ slab is adopted as the substrate. The sensitivity increases with the decrease of the MKR ring diameter. The achieved maximum magnetic field sensitivity is 277 pm/mT. The results of this work have the potential to promote the development of magnetically controllable optical devices and the design of ultra-compact cost-effective magnetic field sensors.

## 1. Introduction

Recently, the micro-nano fiber (MNF) ring resonator has been intensively investigated for its ability to measure various physical parameters, such as magnetic field, temperature, bio-chemical solution, current, salinity, force, electric field, and refractive index (RI) [1,2,3,4,5,6,7,8]. The advantages of the structure lie in its small size, anti-interference, quick response, high resolution, low detection limit and high sensitivity [9,10,11,12,13,14,15,16]. Among various MNF ring resonators, the microfiber loop resonator (MLR), microfiber knot resonator (MKR) and microfiber coil resonator (MCR) attract particular interest [17]. Compared with the MLR and MCR, MKR is an outstanding MNF ring resonator due to its stable performance and adjustable free spectral range (FSR). Li et al. have demonstrated the all-fiber magnetic field sensor based on MKR and magnetic fluid (MF) with a sensitivity of 3 pm/mT [4]. Amili et al. have designed a magnetically controllable silicon microring with ferrofluid cladding and obtained a magnetic field sensitivity of 1.68 pm/Oe [18].

In this work, a fiber-optic magnetic field sensor based on MKR with MF cladding is proposed and experimentally demonstrated. The MgF_2_ slab with low RI is used as the substrate to support the MKR, which will result in the large evanescent field of the MKR accessing the MF cladding. Therefore, the transmission spectrum of the MKR is highly sensitive to the external RI. The sensitivity is greatly improved. In addition, the proposed MKR sensor has the potential to be utilized in some harsh conditions, such as in narrow gaps and remote monitoring.

## 2. Fabrication and Sensing Principle

To fabricate the MKR, the single-mode fiber is tapered into microfiber with the flame-heated taper-drawing technique [19]. The diameter of the as-fabricated microfiber is 4 μm. Then, the microfiber is knotted to obtain the MKR with desired ring diameter. The MKR is placed on the low-index MgF_2_ slab and fixed with UV glue at the non-tapered area. Finally, the MKR with MgF_2_ substrate is inserted into a glass cell filled with MF. The employed MF is a water-based MF with a density of 1.06 g/cm^3^ at 25 °C, which is provided by Beijing Sunrise Ferrofluid Technological Co., Ltd., Beijing, China. The diameter of the magnetic nanoparticles is around 10 nm. The RI of MgF_2_ slab is ~1.37, which is much smaller than that of microfiber. This will avoid the leaking of MKR evanescent field into the substrate. Therefore, most of the MKR evanescent field can penetrate into the surrounding medium (MF). Thus, the sensitivity of the structure will be improved.

**Figure 1:**
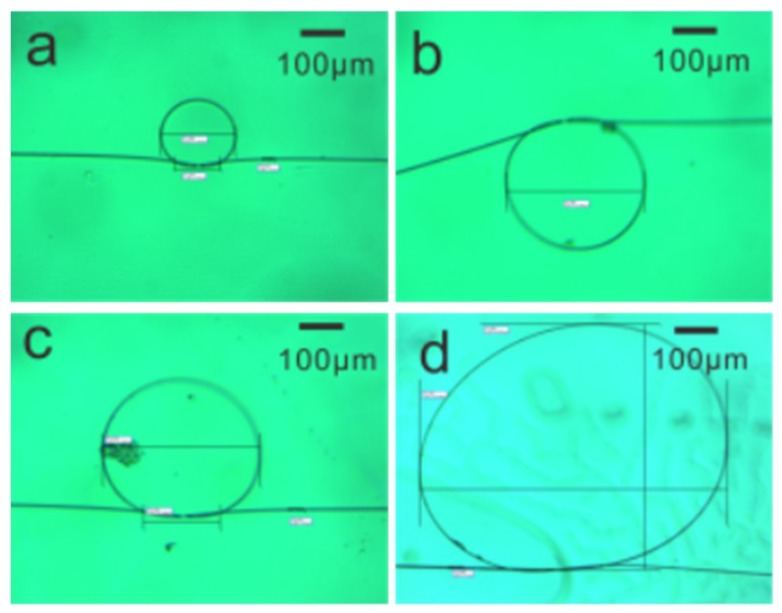
Optical micrographs of the as-fabricated microfiber knot resonators (MKRs) with ring diameters of 155 μm (**a**), 289 μm (**b**), 328 μm (**c**), and 594 μm (**d**).

The optical micrographs of the as-fabricated MKRs are shown in Figure 1. The ring diameters D are 155, 289, 328 and 594 μm, respectively. There is a slight deviation from an absolutely perfect circle for the as-fabricated structures, especially for those with large diameters.

For the MKR, the resonance wavelength is expressed as [20]

(1) $$\lambda_{res}=\frac{2\pi n_{eff}L}{m}$$

where $n_{eff}$ and L are the effective RI and circumference of the knot, respectively. m is the resonance order. It is obvious from Equation (1) that the resonance wavelength $\lambda_{res}$ changes with $n_{eff}$. As the RI of MF increases with the magnetic field (usually around 0.0001 RIU/Oe) [21,22,23,24], the effective RI of knot will also change with the magnetic field. Therefore, the resonance wavelength will shift with the magnetic field. Magnetic field sensing is realized by monitoring the resonance wavelength shift.

## 3. Experiments and Discussion

**Figure 2:**
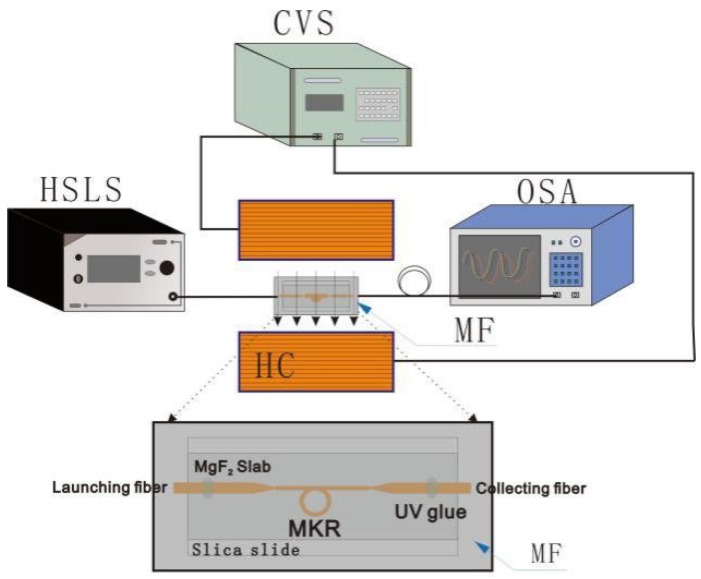
Schematic of the experimental setup for investigating the sensing properties.

The experimental setup for investigating the sensing properties is shown in Figure 2. Light from the highly stabilized laser source (HSLS) is launched into the sensing structure, and the output light is detected and analyzed by an optical spectrum analyzer (OSA, Yokogawa AQ6370C, Tokyo, Japan). The MKR structure under test is placed between two coils in a Helmholtz configuration (HC). The current-voltage source (CVS) provides electric current which flows through the coils, generating an adjustable uniform magnetic field. The magnetic field direction is parallel to the MKR plane. During our experiments, the ambient temperature is kept constant.

**Figure 3:**
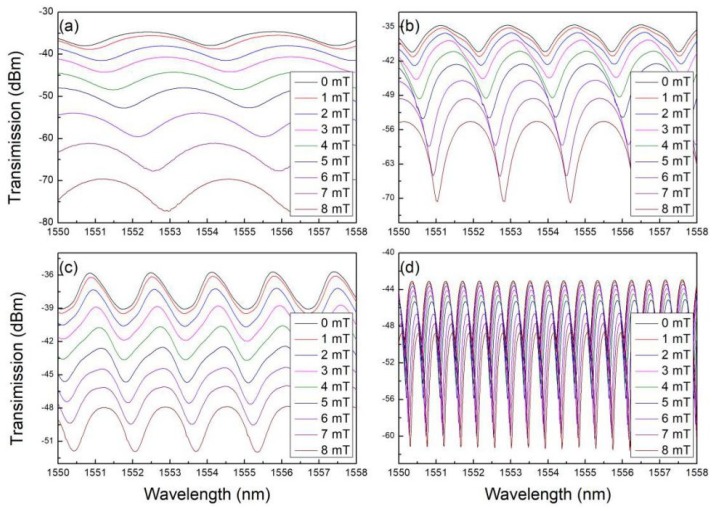
Transmission spectra of the MKRs at various magnetic fields. The ring diameters of the MKRs are 155 μm (**a**), 289 μm (**b**), 328 μm (**c**), and 594 μm (**d**).

Figure 3 shows the transmission spectra of the MKRs at various applied magnetic fields. For all the MKRs, the resonance dip red-shifts with the external magnetic field. Theoretically, the shift for *m*-th order resonance wavelength can be derived from Equation (1),

(2) $$\Delta \lambda_{res}=\frac{\partial n_{eff}}{\partial n_{MF}}\frac{\partial n_{MF}}{\partial H}\frac{\Delta H}{n_{eff}}\lambda_{res}$$

where ΔH is the change of magnetic field intensity. As $n_{eff}$ increases with $n_{MF}$, $\frac{\partial n_{eff}}{\partial n_{MF}}>0$. The relationship between RI of MF and magnetic field intensity meets the Langevin function [25], so $\frac{\partial n_{MF}}{\partial H}>0$. As $n_{eff}$ increases with $n_{MF}$, $\frac{\partial n_{eff}}{\partial n_{MF}}>0$. Consequently, the resonance spectrum red-shifts with the magnetic field. In addition, the extinction ratio of the transmission spectra increases with the magnetic field for a certain MKR structure, which may be assigned to the power coupling change of the structures.

**Figure 4:**
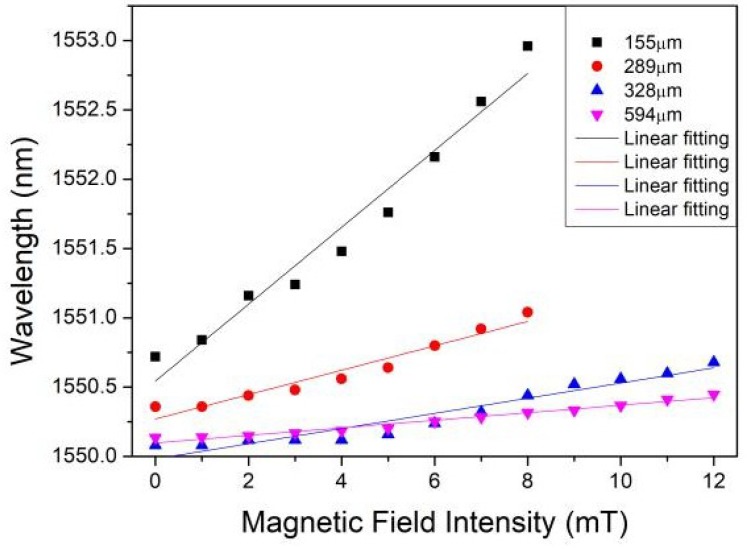
Wavelength shift as a function of magnetic field for the MKR sensors. The ring diameters of the MKRs are 155 μm (**a**), 289 μm (**b**), 328μm (**c**), and 594 μm (**d**).

**Figure 5:**
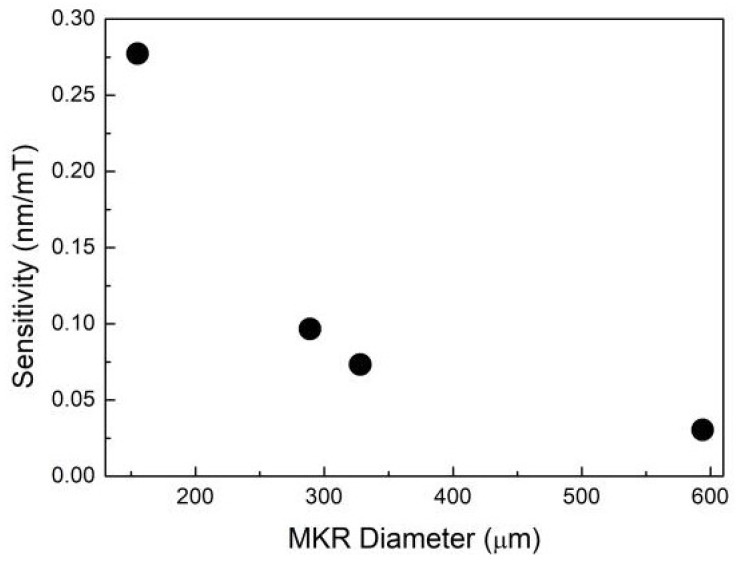
Magnetic field sensitivity of the MKR sensors as a function of the MKR ring diameter.

The magnetic-field-dependent wavelength shift is further plotted in Figure 4. Figure 4 reveals that the magnetic field sensitivities are about 277, 97, 73 and 30 pm/mT, respectively. Figure 5 explicitly displays the magnetic field sensitivity as a function of the MKR ring diameter. As the diameter of MKR ring decreases, the sensitivity increases monotonously. The experimental data slightly deviate from linearity, which may contribute to the non-circular structures.

For the microfiber with fixed diameter, the evanescent field increases with the decrease of bending radius. Therefore, the evanescent field of MKR enhances with the decrease of the MKR ring diameter. This will lead to higher sensitivity for an MKR with smaller diameter.

Table 1 compares the sensitivity of various fiber-optic magnetic field sensing structures, which shows that the achieved sensitivity of our structure is relatively high. The sensitivity of our work is around 16 times higher than that of the silicon microring [18] and is the same order of magnitude as that of a microstructured polymer optical fiber structure [26]. We would like to further point out that the RI of MF only depends on the absolute value of the magnetic field. Therefore, the proposed sensor cannot determine the sign of the magnetic field. Besides this, the polarization of incident light and relative orientation between the magnetic field and MKR plane will affect the sensing properties [22,27].

Considering the 0.02 nm wavelength resolution of traditionally commercial OSA, the magnetic field sensing accuracy can reach 0.07 mT, which can be further enhanced by using a demodulator with higher resolution.

## 4. Conclusions

In conclusion, the MKR combined MF is proposed for magnetic field sensing. The resonance wavelength varies approximately linearly with the applied magnetic field. The obtained magnetic field sensitivity is 277 pm/mT for the MKR with a ring diameter of 155 nm. As the diameter of MKR decreases, the sensitivity of the structure increases correspondingly. The sensor designed in this paper can adjust the Q value and sensitivity by micro-operation. It is also easy to fabricate and integrate with traditional optical fibers, and it can be applied to a variety of microphotonic devices.

## Figures and Tables

**Table 1:** Sensitivity comparison of various magnetic field sensing structures.

Number	Structure	Sensitivity	Reference
1	MKR (silica gel)	3 pm/mT (0.3 pm/Gs)	[4]
2	Silicon microring	1.68 pm/Oe	[18]
3	Taper-like and lateral-offset fusion splicing	26 pm/Oe	[28]
4	Fabry–Perot interferometer	44 pm/mT (4.4 pm/Gs)	[29]
5	Taper microstructured fiber	117.9 pm/mT (11.79 pm/Gs)	[30]
6	MKR (MgF_2_ substrate)	277 pm/mT (27.7 pm/Gs)	This work
